# Annotations

**Published:** 1897-05-08

**Authors:** 


### ANNOTATIONS.

### The Adelaide Hospital, South Australia.

THE discord which reigns in the profession at Adelaide does not seem likely to be lessened by the replies given by Dr. Leith Napier and Dr. Ramsay Smith to the charges brought against them during the inquiry into the affairs of the hospital. As an answer to these charges their letters are effective enough, for truly they smite their enemies hip and thigh. At the same time we cannot but regret that such letters should have to be written, and that the customs of the country should sanction their appearance in the public Press. Among the many errors committed in the conduct of this affair not the least was the failure of the Government to recognise the unanimity of the profession in the Colony. Perhaps if they had done so they would have been less ready to part with their old officers. But when they determined to have a change it was not enough to import two new men. In the interests of the patients a completely new staff was essential. This, we believe, they have now obtained, but in the interval difficulties naturally arose, and we are quite prepared to believe the patients suffered. It can never be well that the members of the staff of any hospital should be at daggers drawn—a moral which applies, we are afraid, to hospitals nearer home than Adelaide.

### The Pollution of Rivers.

IN a presidential address, recently delivered before the Philosophical Society of Glasgow by Professor Glaister, attention has been called to the continued pollution of Scottish rivers, and we have no doubt that much that he says about the condition of rivers north of the border is applicable to English rivers also. It is to be noted that in Scotland, as in England, many of the forms of river pollution which are found most difficult to deal with are those which arise from manufacturing processes. That form of fouling of streams which results from the discharge into them of crude sewage derived from residential towns and villages has characters which are fairly uniform, and the methods by which it can be prevented are now pretty well understood. Trade pollution, however, stands on a very different footing. Every trade produces a different effluent, and, until it is demonstrated that it is capable of purification, each manufacturer maintains that such a thing is impossible. Even then he is too ready to assert that to purify it means ruin to his trade, and to refuse to do anything until prodded by the bayonet of the law. It is only lately that we have fully understood the especial evils that are connected with the discharge of trade effluents into rivers. Many a time have chemical manufacturers asserted that their effluents were really advantageous from their disinfectant action upon ordinary domestic sewage. We know now, however, that the way in which rivers and streams purify themselves of sewage is by the growth of micro-organisms in the organic matter contained therein both while being carried along by the stream and when deposited upon its banks, and we also know that so far from chemicals and trade refuse ever being a benefit to any river, they are an injury, by killing those micro-organisms on which its restoration to purity depends. It is interesting to observe that where the bayonet of the law does prod with sufficient vigour manufacturers do find a way, and that the most difficult effluents do become pure when injunctions and other processes of law are brought to bear upon those who produce them. Of this Professor Glaister gives several examples, showing also how in some instances the value of the waste products recovered has been more than sufficient to cover the expense of the necessary works.

### Death in the Bath.

THE unfortunate death of an elderly man in a Turkish bath a few days ago draws attention to the elements of danger and of safety in the administration of baths of this description. The essential peculiarity of the Turkish, as distinct from all other forms of baths, is the very much higher temperature to which the bather is subjected. In the hottest room the temperature may be from 250 deg. to 300 deg. F., that is, about the temperature at which an oven is kept for the baking of puff pastry, and considerably higher than is required for ordinary cakes or for baking meat. Under these circumstances the safety of the bather depends on two things—his power of perspiring and the power possessed by the air in the bath of evaporating his perspiration. To take the latter first, it must never be forgotten that the Turkish bath is not a vapour bath. It is a hot-air bath, and the life of the bather depends on the air remaining far from saturated with watery vapour. If in consequence of any deficiency of ventilation this hot air were to become saturated with moisture, evaporation from the bather's skin would cease, and he would be steamed alive. His safety depends absolutely and entirely on evaporation from the surface of the skin and the bronchial mucous membrane. This being so, it is obvious that the bather will suffer distress, and, in fact, be affected with partial heat stroke, even when the ventilation is good, if his own capacity for perspiration is diminished. The respiration of some persons is affected in a reflex manner by the application of heat to the bronchial mucous membrane, much as it is by the breathing of certain medicated vapours, so that on entering the bath a form of suffocation is sometimes experienced which has nothing to do with either body heat or heart strain. In the majority of cases, however, distress, if it occur, is due either to defective power of perspiring or, much more commonly, to strain upon the heart. It is for this reason that Turkish baths are so often dangerous to those who suffer from organic heart disease. Where a comparatively sound heart labours to drive impure blood through contracted blood-vessels, we may well imagine that the opening of the cutaneous circulation by a Turkish bath may actually lighten the load upon it. But where the heart itself is feeble and ts valves imperfect it is incapable of acting efficiently at the greater speed required, and sudden failure may result. Besides this, it must be remembered that a Turkish bath is a gymnastic exercise to the blood-vessels as well as to the heart, and thus we come to the generalisation that, while the Turkish bath is a harmless luxury to the man with a good heart and elastic vessels, it is a dangerous performance to one whose heart and blood-vessels are defective, unless far more than ordinary care is taken.

